# Caregivers' Knowledge, Attitude, and Practice towards Pressure Injuries in Spinal Cord Injury at Rehabilitation Center in Bangladesh

**DOI:** 10.1155/2022/8642900

**Published:** 2022-06-14

**Authors:** Niraj Singh Tharu, Monzurul Alam, Shristi Bajracharya, Gautam Prasad Chaudhary, Jitendra Pandey, Mohammad A. Kabir

**Affiliations:** ^1^Faculty of Medicine, Dhaka University, Dhaka, Bangladesh; ^2^Department of Biomedical Engineering, The Hong Kong Polytechnic University, Hong Kong, China; ^3^Department of Pharmacy, Crimson College of Technology, Pokhara University, Butwal, Nepal; ^4^Department of Statistics, Jahangirnagar University, Dhaka, Bangladesh

## Abstract

The purpose of this study was to determine caregivers' knowledge, attitude, and practice (KAP) on the prevention and care of pressure injuries (PIs) in individuals with spinal cord injury. A quantitative cross-sectional study with descriptive correlation design was used to implement a modified semistructured questionnaire using a convenient sampling method. McDonald's standard of learning outcome measurement criteria was used to categorize caregivers' KAP. A Pearson product-moment correlation coefficient (*r*) was utilized to assess the relationships between caregivers' KAP, with a *p* value of 0.05 or less considered statistically significant. The study findings indicate that caregivers had a moderate level of knowledge (M = 73.68%, SD = 6.43), a neutral attitude (M = 70.32%, SD = 6.89), and a moderate level of practice (M = 74.77%, SD = 9.08). A positive correlation existed between caregivers' knowledge and attitude (*r* = 0.30, *p* < 0.01), as well as between knowledge and practice (*r* = 0.37, *p* < 0.01). Nevertheless, there was no correlation between attitude and practice (*r* = 0.12, *p* > 0.05). The study findings suggest that caregivers need to develop a positive attitude and expand their knowledge in order to improve their practice. The KAP factors that require higher priority were positioning and turning the patient, preventing skin breakdown, assessing weight changes over time, interest in patient care, additional care for PIs, frequently changing the individual's position, priority to PI care, interest in other types of care other than PIs, using special cushions, consulting doctors on a regular basis, being aware of clothing and fabrics, proper transfer technique, pressure relief, and skin inspection, among others.

## 1. Introduction

Spinal cord injury (SCI) is a neurological damage that results in motor and sensory disturbances, as well as difficulty performing daily living tasks [1]. Each year, the worldwide incidence of SCI ranges from 8.0 to 246.0, whereas the prevalence ranges from 236.0 to 1298.0 per million people. However, when looking at statistics from the last decade, a huge increase has been noticed [2]. Individuals with SCI are susceptible to secondary complications that need massive health care services [3]. People with SCI experience immobility and rely on external help to do daily tasks, with restricted mobility being the most prevalent cause of pressure injuries (PIs) [4]. It was shown that the development of PIs during inpatient rehabilitation has the highest chances of recurrence. As a result, avoiding PIs will almost certainly result in a decrease in the burden on people with SCI and the community [5].

Due to their limited mobility and impaired sensations during their lifetime, individuals with SCI are extremely vulnerable to PIs [6]. The PIs are a frequent complication of SCI, and their treatment costs seem to be 2.5 times higher than those of preventive measures, prolonging the infection period and necessitating extended treatment, which may potentially result in death [7]. PIs affect approximately 30–85% of people with SCI at some point in their lives, with the remaining 15% developing PIs later in life [8].

It was found that 28% of individuals with SCI admitted to the Centre for the Rehabilitation of the Paralyzed (CRP) developed PIs [9]. Even after discharge from the hospital, SCI sufferers develop several complications, and PIs seem to be one of the most common complications [10]. A study conducted in Bangladesh showed that, upon completing rehabilitation and following discharge, 56.4% of SCI individuals died within five years, whereas only about 16.4% survived longer than ten years, with the majority of deaths occurring at home [11]. In contrast, another study reported that one in every five SCI people who are wheelchair-bound die within two years of discharge from the hospital, with the primary cause of death being PIs [12]. There remains a gap, considering that a lack of appropriate knowledge, attitude, and practice among caregivers could be one of the reasons for developing pressure ulcers, which could be filled through this study.

Globally, caregiving is estimated to rise in demand as people live longer mostly with chronic diseases [13]. Nowadays, caregivers offer a variety of patient care services that were formerly provided by health care experts in the past [14], making a significant contribution to the lives of patients, even at the expense of their own health and wellbeing [15]. Caregivers offer primary assistance to SCI individuals in doing everyday activities, and studies have shown that having appropriate knowledge of PI management aids in the patient's rapid recovery [4]. The treatment of PIs is very challenging, even for health experts; instead of having knowledge of PIs, they are attempting to minimize the rate of PI development [16, 17]. Therefore, caregiver assistance is essential when regular follow-up visits by health experts are restricted due to a lack of advanced health care institutions and resources [18]. A study revealed that that caregivers' inability to provide proper patient care resulted in the development of PIs and immobility in individuals with SCI during the acute stages of the injury [14]. In contrast, caregivers' involvement in patient care contributes to the reduction of secondary complications and the maintenance of physical health in people with SCI [19]. The time and effort required to complete daily living tasks by people with SCI has a detrimental effect on caregivers' lives [20]. Caregivers providing long-term care expressed a negative attitude towards the disability [21], and since people with tetraplegia need longer care, caregivers' attitude was influenced, resulting in a decrease in care quality [20]. Therefore, a study identified that SCI individuals recognized the need for training regarding secondary complications among caregivers during initial patient rehabilitation and inpatient care [22]. The investigation of bedsore practices revealed that 12% of caregivers used inappropriate bed mobility techniques that worsened the PIs [4] and also lacked adequate knowledge and practice for the prevention of PIs in immobilized patients [23]. Alternatively, when individuals with SCI were interviewed about preventive measures, it was discovered that they could describe just a few preventative approaches for PIs and had very limited knowledge in this area [24]. Nevertheless, if the patients lack sufficient understanding, the caregivers face a comparable risk. In approximately 95.1% of PIs, the condition is preventable with appropriate knowledge. However, insufficient skills might deteriorate the PI condition [25]. Additionally, there is a need for an appropriate knowledge towards prevention and care of PIs in primary caregivers in low- and middle-income countries (LMICs) such as Bangladesh in order to develop effective preventive measures [18]. A large number of studies on caregiving have focused on psychological consequences, social integration, caring duties, interpersonal relationships, etc., and KAP have received less attention [13, 14]. Furthermore, having appropriate KAP among caregivers may provide assistance in reducing economic expenses and health care needs [26]. The objective of this study was to determine the knowledge, attitude, and practice (KAP) of caregivers regarding the prevention and care of PIs in SCI individuals in order to identify areas that require special care. The specific aims of the study were to (i) identify sociodemographic characteristics of the caregiver that affect prevention and care of PIs, (ii) identify the relationship between the caregivers' KAP and sociodemographic variables, and (iii) observe the impact of caregiving depending on the type of injury of the SCI individual. The study focused on empowering caregivers of SCI individuals by recognizing the factors that require additional care and teaching strategies of prevention and care to reduce the recurrence of PIs. The findings of this study may potentially be used to support future development of PI preventive strategies for patient care of individuals with SCI admitted to rehabilitation centers or living in the community.

## 2. Methods

A quantitative cross-sectional study was conducted among caregivers of individuals with SCI admitted for rehabilitation at the CRP in Bangladesh. The study included the caregivers of all SCI people admitted to the CRP after a recent SCI following clinical management. Both the paraplegia and tetraplegia, with or without PIs, were identified, and their caregivers were interviewed. In addition, the caregivers of people with tetraplegia who spent at least one month as the primary caregiver of the SCI individual were questioned, whereas those with paraplegia who spent two weeks at a halfway hostel (discharge phase) were interviewed. The study included a total of 127 caregivers using a convenience sampling method. The questions were adapted from the National Pressure Ulcer Advisory Panel, the European Pressure Ulcer Advisory Panel, and the Pan Pacific Pressure Injury Alliance's 2014 published on Pressure Ulcer Prevention and Treatment (NPUAP/EPUAP/PPPIA, 2014) [27], as well as the Pressure Ulcer Attitude Questionnaire [28]. There is no available outcome measure to assess the assistance to individuals with SCI by the caregivers. Therefore, a modified questionnaire is used [29]; though to ensure the quality of the data, the questionnaire was prepared in an English-Bengali version. Prior to data collection, experts working for SCI rehabilitation reviewed the questionnaire for clarity, comprehensiveness, and content validity, and a pilot test was conducted on 10 caregivers in different units of the SCI department at the CRP to determine its reliability. The study included caregivers with main responsibility for inpatient care who were at least 18 years old. However, caregivers whose family members with SCI were hospitalized for rehabilitation after more than a year of SCI were excluded. The structured modified questionnaire was based on perspectives on SCI in Bangladesh and consisted of four sections: sociodemographic and caregivers' knowledge, attitude, and practice questionnaire regarding PI prevention and care. The sociodemographic questionnaire had 8 questions that assessed the respondent's age, gender, educational status, marriage status, employment status, residence status, living area, and caregiver-patient relationship. Similarly, caregiver knowledge was tested using an 18-item structured questionnaire in which respondents were asked to rate each item as correct, partially correct, or incorrect. The questionnaire had both positive and negative item questions, with negative item scores reversed. Similarly, a 16-item structured questionnaire was used to measure respondents' level of practice, with respondents being asked to identify whether they practiced always, sometimes, or never. The 3-point Likert scale was used to assess both knowledge and practice, and the potential total score was converted to a percentage, with higher scores indicating a better level of knowledge and practice. The mean (*M*) and standard deviation (SD) were calculated for categorization. A *p* value of 0.05 or less was considered statistically significant. McDonald's standard of learning outcome measurement criteria [30, 31] was used to categorize caregivers' knowledge and practice about PI prevention and care. The criterion was divided into five categories: very low (˂60%), low (60%–69.99%), moderate (70%–79.99%), high (80%–89.99%), and very high (90%–100%). Again, caregiver attitude was assessed using a 12-item structured questionnaire in which respondents rated five levels of attitude: strongly agree, agree, neither agree nor disagree, disagree, and strongly disagree. There were questions with both positive and negative items, and negative item scores were reversed. On the basis of mean percentage and standard deviation, the total score of attitudes was categorized into three levels: negative attitude if below (mean ± SD), neutral attitude if at (mean ± SD), and positive attitude if above (mean ± SD). Simultaneously, the Pearson product-moment correlation coefficient (*r*) was used to examine the relationships between the levels of caregivers' knowledge, attitude, and practice.

## 3. Results

The mean age of the caregivers (Table 1) was 38.21 years (SD = 11.95), with a minimum and maximum age of 18 and 72 years, respectively. The majority of the caregivers (29.1%) were aged between 38 and 47 years, while the least aged group of the caregivers (4.7%) fell into the category of 57 years and above. Most of them were female (76.4%), and the caregivers with no formal education occupied the highest proportion (37.0%) who were engaged in taking care of the SCI individuals, while the caregivers with higher educational status as graduates and above were only 5.5%. Many of the caregivers were married (86.6%), and among them, nearly two-thirds of the caregivers were housewives (69.3%). The residence status of the caregivers revealed that 82.7% of the caregivers lived in the same house with the SCI individual and 81.9% were from the rural area, where the wife (40.9%) and mother (19.7%) were the primary caregivers. On the other hand, the mean age of the person with SCI (Table 2) was 37.97 years (SD = 13.76) and the duration of SCI was 4.76 months (SD = 1.33). The higher proportion of care recipients were males (82.7%), where accidents (79.5%) seem to be the most common cause of injury. Similarly, 26.8% of SCI individuals developed PIs, while 64.6% were with paraplegia, followed by 35.4% with severe SCI (tetraplegia).

**Figure 1:**
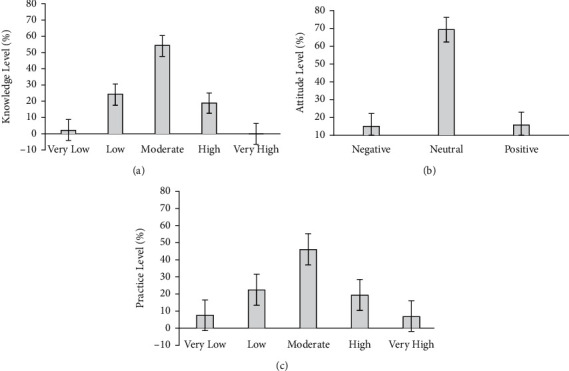
Caregivers' (a) knowledge and (c) practice regarding prevention and care of pressure injuries (PIs) based on McDonald's standard of learning outcome measure criteria: very low (<60%), low (60–69.99%), moderate (70–79.99%), high (80–89.99%), and very high (90–100%). (b) Caregivers' attitude regarding prevention and care of PIs, categorized as follows: negative, below (mean ± 1 SD); neutral, at (mean ± 1 SD); and positive, above (mean ± 1 SD).

The caregiver's knowledge regarding prevention and care of PIs was at a moderate level (*M* = 73.68%, SD = 6.43) with minimum and maximum scores of 55.56% and 87.04%, respectively. As shown in Figure 1(a), it was found that 26.8% of caregivers possessed a very low (2.4%) to low (24.4%) level of knowledge. The majority of caregivers (54.3%) had a moderate level of knowledge, whereas 18.9% scored a high level of knowledge, and no caregiver had a very high level of knowledge regarding the prevention and care of PIs. The caregivers mostly lacked the knowledge to safely position and turn the patient, prevent skin breakdown, and assess weight changes over time, among others.  Figure 1(b) shows that the caregiver's attitude regarding prevention and care of PIs was at a neutral level (*M* = 70.32%, SD = 6.89) with minimum and maximum scores of 51.67% and 93.33%, respectively. The scoring criteria based on mean ± SD were categorized as negative (<63.43%), neutral (63.43%–77.21%), and positive (>77.21%). Most of the caregivers (69.3%) achieved a neutral level of overall attitude towards prevention and care of PIs, while 15.0% of the caregivers showed a negative attitude. Moreover, 15.7% of the caregivers had an overall positive attitude towards prevention and care of PIs. The factors with the most negative attitudes were those relating to patient care, additional care for PIs, changing the position of a patient frequently, priority to PIs, requiring lots of time, and interest in other care than PIs, among others. The caregiver's practice regarding prevention and care of PIs was at a moderate level (*M* = 74.77%, SD = 9.08) with minimum and maximum scores of 54.17% and 93.75%, respectively. As shown in Figure 1(c), it was found that nearly half (45.7%) of the caregivers scored moderate level and 18.9% of the caregivers had a high level of practice regarding prevention and care of PIs. However, it was observed that 29.1% of caregivers possessed a very low (7.1%) to low (22.0%) level of practice. The low-practice factors included the use of special cushions, consulting doctors on a regular basis, being aware of clothing and fabrics, proper transfer technique, pressure relief, and skin inspection, among others.

The relationship between KAP (Table 3) was calculated using correlation analysis that revealed a positive correlation between caregivers' knowledge and attitude (*r* = 0.30,  *p* < 0.01) and between knowledge and practice (*r* = 0.37,  *p* < 0.01). However, there was no correlation between attitude and practice (*r* = 0.12,  *p* < 0.05) regarding prevention and care of PIs. Moreover, the knowledge and sociodemographic characteristics of caregivers (Table 4) indicated a significant relationship with age (*p* < 0.001), educational status (*p* < 0.001), and caregiver-patient relationship (*p* < 0.01), whereas attitude demonstrated a significant relationship with age (*p* < 0.05), educational status (*p* < 0.001), and occupational status (*p* < 0.01). Additionally, practice had a significant effect on gender (*p* < 0.05), educational position (*p* < 0.001), and the caregiver-patient relationship (*p* < 0.05). This shows that sociodemographic characteristics associated with KAP may have an effect on the caregivers' service. On the other hand, the relationship between sociodemographic characteristics of individuals with SCI and KAP of caregivers (Table 4) revealed that duration of injury (*p* < 0.01) showed a significant relationship with the caregivers' knowledge, while duration of SCI (*p* < 0.05) and SCI person with or without PIs (*p* < 0.01) demonstrated a correlation with attitude. Moreover, duration of SCI (*p* < 0.001), SCI person with or without PIs (*p* < 0.001), and care recipient with paraplegia or tetraplegia demonstrated a correlation between practice of caregivers and people with SCI. The findings indicate that the duration of injury could affect the caregiver's KAP. Also, presence of PIs in care recipients could influence caregivers' attitude and practice. Again, the practice of caregivers could vary depending on type of injury, i.e., paraplegia or tetraplegia.

## 4. Discussion

### 4.1. Sociodemographic Characteristics

The study results showed that a higher proportion of caregivers were female and in the middle-aged group (38–47 years). It indicates that, in LMICs such as Bangladesh, females are prioritized when it comes to caring for anyone in the family. As shown in previous studies, women are often assigned to care for people with disabilities as a consequence of their traditional role as caregivers for the house and family [32] and spouses were primarily involved [13]. With increasing educational levels, the proportion of caretakers decreased. This demonstrates clearly that educated caregivers were uninterested in the role of caregiver. In terms of occupation, it was shown that majority of the caregivers were housewives. Men are more likely to be exposed to employment or activities that put them at risk of SCI, while women often work inside the home and stay indoors [11]. Relatives (kins/cousins) were also found to be primary caregivers, and the spouses of male SCI people, as well as the mothers of female SCI individuals, were primarily active in caretaking among family members, if the individual was married. A study on various aspects of caregivers' lives showed age and educational status have a significant relationship among them [26]. In contrast, caregivers' educational attainment varied considerably in terms of proportions at the higher education level [33].

### 4.2. Level of Knowledge

The study results revealed that caregivers had a moderate level of knowledge, i.e., neither high nor low, indicating a lack of information on PI prevention and care. A study reported that caregivers' understanding of bedsore care was insufficient, and their practices were found to be incorrect [34]. Moreover, caregivers lacked appropriate knowledge on the prevention of immobility-related problems [23]. It is assumed that age and educational background may play a role in this moderate level of knowledge. However, the findings showed a very significant relationship between age and level of education. It is likely that caregivers with a higher level of education have more knowledge than those with a lower level of education [4]. Additionally, more than three-quarters of responders are between the ages of 18 and 27 years and between 28 and 37 years. Considering that this is an age of education and learning, the moderate level of knowledge may be a result of this. In the literature, age had a significant effect on knowledge scores, with the elderly demonstrating superior knowledge [35]. The majority of caregivers are housewives, and they have some understanding of basic care but lack specific knowledge about transfer and positioning. They seemed to be more familiar with the various aspects of general care than with PI care. Mersal reported that there is a significant correlation between knowledge level, age, sex, marital status, and kin relationships [4]. In terms of the relationship between caregiver and patient, it is predicted that if the patient is married, the wife and mother would be the primary caregivers, with other family members rarely involved. The associated finding is that respondents' levels of knowledge are statistically significant in relation to their area of residence, marital status, and educational status [23].

### 4.3. Level of Attitude

The findings indicated that a greater proportion of caregivers had a neutral attitude towards PI prevention and care, implying that caregivers were either unaware of PI prevention and care or had no understanding of how to avoid PI development. It is predicted that there is a relationship between age and attitude. In contrast, additional analysis supported this statement. Age and attitude were shown to be significantly correlated. Females exhibited a more positive attitude than males, whereas housewives showed a more positive attitude than other occupations [35]. On the other hand, caregiving for an individual with SCI requires much more than assistance with daily living activities and may have both positive and negative effects on the caregiver [29]. It was observed that a large percentage of caregivers were in the age range of 38–47 years; as members of the middle age group, it is possible that they were aware of the disability and were determined to accept the consequences resulting from it. As a result, they may have had a neutral attitude towards PI prevention and care. The caregiver has an impact on the care provided to the recipient [34], though, for appropriate patient care, the caregiver role must be positive [15]. Similarly, compared with other occupations, a high proportion of housewives had a neutral attitude towards PI prevention and care. This might be because they did not have other duties aside from being a caregiver, such as office work or family responsibilities. Additionally, as housewives, women perform everyday household chores that are remarkably comparable to those of caregivers. Family members are the primary caregivers for the majority of the time when a patient needs care [34]. Regarding the relationship between caregiver and patient, it also revealed a strong correlation with the attitude score. The wives of SCI individuals had a more neutral attitude towards preventing and caring for PIs than other family members. This might be related to their relationship since it was noticed that married people with SCI prioritized their wife and mother when it came to caregiving. This might be a reason enough to show a neutral attitude rather than a negative attitude. The caregiver's relationship and bonding with the person with SCI reflects their common interest in caregiving [35]. It has been demonstrated that spouses of individuals with SCI who express a positive attitude towards caring have improved emotional wellbeing. Additional studies on the positive features of caring are thus necessary in the future [26].

### 4.4. Level of Practice

The caregiver's practice regarding prevention and care of PIs was determined to be at a moderate level. The levels of knowledge and practice were equivalent to each other. In this study, caregivers' practice was reflected by their knowledge. 95.1% of PIs are preventable with the knowledge of their risk factors [25]. Gender had a significant relationship with practice level. A probable explanation for this moderate level of practice across genders is that females are mostly involved in household chores since the majority of caregivers were found to be housewives. This enables them to provide a relatively moderate level of care to SCI individuals compared with male caregivers. The findings of our study were found to be different on various aspects in relation to the studies of nurses [36, 37] and other health care professionals [25]. Previous studies indicate that education and the sharing of standardized information are the most successful strategies for enhancing the caregiver's knowledge and performance, even more so when programs involve the care receiver [38]. Educational status is a factor associated with a moderate level of practice. It has been shown that caregivers with a higher level of education demonstrate a higher level of practice. A similar significant correlation was found between practice level, age, gender, marital status, and kin relationships [4]. Similarly, the academic status of caregivers had an effect on the quality of care [32].

### 4.5. Relationships between Knowledge, Attitude, and Practice

The study discovered a moderately significant relationship between knowledge and attitude regarding PI prevention and care. According to the KAP model, a factor that could affect attitude is specific knowledge. Following that, the KAP model is supported by the findings of this study. This might be because caregivers' attitude was influenced by their age, educational status, and the relationship between caregiver and patient. A study conducted in Pakistan found a significant relationship between inadequate or inappropriate knowledge and the development of PIs, where the level of knowledge was determined by the participants' training and occupation. Meanwhile, both attitude and practice were shown to be important predictors of improved knowledge [17]. It revealed that caregivers who were the SCI individual's wife and were in an active period of life demonstrated positive attitudes, as did caregivers who had a higher education. Thus, knowledge in itself has an impact on the caregiver's attitude development. Individuals' knowledge and attitude may have an effect on their practice, according to the KAP model. Caregivers are needed to adapt to changing conditions and readjust their attitude and practice for effective caregiving [14]. In general, social, psychological, and physical factors all affect the lives of individuals with disabilities as well as their families. Nowadays, the rehabilitation concept has shifted towards a more patient-centered approach. There is evidence to suggest that including patients and caregivers in a study may improve its quality and ensure its applicability and usefulness [33]. In this regard, caregivers need additional continuing education and training programs on PI prevention and care that may influence positive attitudes and eventually result in successful PI prevention and care practice. Previous studies discovered a positive correlation between knowledge and attitude, but not between knowledge and practice. While having a high level of knowledge indicates a good attitude, there was no correlation between knowledge and practice scores [39]. There was a moderate, but statistically significant, positive correlation between caregivers' knowledge and practice regarding PI prevention and care.

These results provide support to the KAP model, which states that knowledge influences practice. A study revealed that caregivers lacked appropriate knowledge and performance skills, although training and educational programs enhance caregivers' knowledge and practice [4]. There was, however, a small and nonsignificant relationship between attitude and practice towards PI prevention and care. The KAP model suggests that, if attitudes develop, they would reflect on practice. As a result, caregivers' practice was not reflected in this study by their attitude. Alternatively, in comparison with standard treatment, electrical stimulation of paralyzed muscles has been demonstrated to significantly enhance paralyzed muscle mass, increase muscle tissue circulation, and ease seated air pressure; and thus, ES may be an effective approach for reducing the occurrence of PIs [5].

It is recommended that to improve caregivers' practice, they should have updated knowledge and information on the prevention and care of PIs. It requires special attention to improve the support systems for people with SCI in Bangladesh during the acute rehabilitation and reintegration periods. Also, the identified factors could be considered while developing programs for PI management in LMICs such as Bangladesh [18]. In this study, 26.8% of individuals with SCI developed pressure ulcer. A study conducted in Bangladesh showed that 28% of individuals with SCI admitted to the Centre for the Rehabilitation of the Paralyzed (CRP) developed pressure ulcer [9]. Therefore, another study conducted in Bangladesh showed that, upon completing rehabilitation and following discharge, 56.4% of SCI individuals died within five years, whereas only about 16.4% survived longer than ten years, with the majority of deaths occurring at home [11]. There remains a gap, considering that a lack of appropriate knowledge, attitude, and practice among caregivers could be one of the reasons for developing pressure ulcers, which could be filled through this study. If caregivers are provided with adequate knowledge, they will be able to manage their stress and develop a positive attitude towards PI care, thus improving their own and the sufferer's quality of life. The study reported on the knowledge, attitude, and practice of caregivers about the prevention and care of PIs in a rehabilitation setting. However, the majority of PIs-related mortality occurs in the home after discharge from the rehabilitation center. In community settings, caregivers' KAP towards PIs may differ. Therefore, it is suggested to conduct the study of KAP among caregivers in the community setting to find out what caregivers think about PI prevention and care in people with SCI after social integration. This way, we can find out more about PI prevention and care at both levels.

## 5. Conclusion

The caregiver's knowledge and practice were at a moderate level, whereas their attitude towards PI prevention and care was at a neutral level. This indicated that caregivers lacked sufficient knowledge and were compelled to provide patient care against their interests; also, their practice was not very satisfactory. There was a positive correlation between caregivers' knowledge and attitude (*r* = 0.30, *p* < 0.01), as well as between knowledge and practice (*r* = 0.37, *p* < 0.01), regarding prevention and care of PIs. In contrast, there was no correlation found between caregivers' attitude and practice towards prevention and care of PIs (*r* = 0.12, *p* < 0.05). This information will be helpful in assisting rehabilitation centers in optimizing their treatment and developing programs based on the experiences of patients and caregivers, as well as aiding health care practitioners for planning patient care during inpatient rehabilitation.

## Figures and Tables

**Table 1:** Sociodemographic characteristics of the caregivers (*n* = 127).

Variables	*n* (%)
Age (years)	
18–27	31 (24.4)
28–37	30 (23.6)
38–47	37 (29.1)
48–57	23 (18.1)
57 and above	6 (4.7)

Gender	
Male	30 (23.6)
Female	97 (76.4)

Educational status	
No formal education	47 (37.0)
Primary education	32 (25.2)
Secondary education	24 (18.9)
Higher secondary education	17 (13.4)
Graduate and above	7 (5.5)

Marital status	
Unmarried	15 (11.8)
Married	110 (86.6)
Widowed	2 (1.6)
Separated	0 (0.0)

Caregiver residence status	
Lives together with patient	105 (82.7)
Lives separately from patient	22 (17.3)

Occupational status	
Housewife	88 (69.3)
Agriculture	13 (10.2)
Service	8 (6.3)
Business	2 (1.6)
Student	8 (6.3)
Factory worker	4 (3.1)
Others	4 (3.1)

Living area	
Rural	104 (81.9)
Semiurban	2 (1.6)
Urban	(16.5)

Relationship between caregiver and patient	
Mother	25 (19.7)
Father	7 (5.5)
Wife	52 (40.9)
Husband	2 (1.6)
Sister	8 (6.3)
Brother	15 (11.8)
Others	18 (14.2)

*n* = number of respondents.

**Table 2:** Sociodemographic characteristics of individuals with SCI.

Variables	Frequency
	Total (*n* = 127)
Age, years, mean (SD)	37.97 (13.76)

Months since SCI, mean (SD)	4.76 (1.33)

Gender, *n* (%)	
Male	105 (82.7)
Female	22 (17.3)

Cause of SCI, *n* (%)	
Accident	101 (79.5)
Illness	12 (9.4)
Other	14 (11.0)

Pressure injuries	
Yes	34 (26.8)
No	93 (73.2)

Type of SCI, *n* (%)	
Paraplegia	82 (64.6)
Tetraplegia	45 (35.4)

*n* = number of respondents.

**Table 3:** Pearson correlation coefficients showing the relationship between caregivers' KAP regarding prevention and care of PIs.

	Knowledge	Attitude	Practice
Knowledge	1.00		
Attitude	0.30^*∗*^	1.00	
Practice	0.37^*∗∗*^	0.12	1.00

^*∗*^ *p* < 0.05, ^*∗∗*^*p* < 0.01, and ^*∗∗∗*^*p* < 0.001 (*p* ≤ 0.05, statistically significant).

**Table 4:** The relationship between caregivers' KAP and sociodemographic characteristics of the caregivers and individuals with SCI in terms of PI prevention and care.

Variables	Knowledge	Attitude	Practice
	*χ* ^2^ *p*	*χ* ^2^ *p*	*χ* ^2^ *p*
Sociodemographic characteristics of caregiver			
Age	43.607^*∗∗∗*^	17.615^*∗*^	9.726
Gender	2.534	4.719	10.147^*∗*^
Educational status	51.316^*∗∗∗*^	40.511^*∗∗∗*^	78.355^*∗∗∗*^
Marital status	38.103	44.133	40.955
Caregiver residence status	30.891	42.861	30.863
Occupational status	15.021	27.061^*∗*^	22.838
Living area	37.772	30.911	39.638
Relationship between caregiver and patient	32.543^*∗∗*^	24.538	35.888^*∗*^

Sociodemographic characteristics of person with SCI			
Age	70.959	61.817	35.568
Months since SCI	25.242^*∗∗*^	28.304	23.478^*∗∗∗*^
Gender	16.574	18.319	19.578
Cause of SCI	42.843	39.823	32.455^*∗∗∗*^
Pressure injuries	20.051	20.369	20.164
Type of SCI	24.019	26.286	23.369^*∗*^

Pearson chi-square = *χ*^2^, ^*∗*^*p* < 0.05, ^*∗∗*^*p* < 0.01, and ^*∗∗∗*^*p* < 0.001 (*p* < 0.05, statistically significant).

## Data Availability

The data of this article are available in the DSpace repository of the CRP library under MSc in Rehabilitation Science, which is publicly available at http://www.library.crp-bangladesh.org:8080/xmlui/handle/123456789/333.
